# An electrochemical sensing platform based on a modified carbon paste electrode with graphene/Co_3_O_4_ nanocomposite for sensitive propranolol determination

**DOI:** 10.5599/admet.1705

**Published:** 2023-03-15

**Authors:** Parisa Karami-Kolmoti, Reza Zaimbashi

**Affiliations:** 1 Department of Chemistry, Graduate University of Advanced Technology, Kerman, Iran; 2 Environment Department, Institute of Science and High Technology and Environmental Sciences, Graduate University of Advanced Technology, Kerman, Iran

**Keywords:** Carbon paste electrodes, graphene/Co_3_O_4_ nanocomposite, differential pulse voltammetry, propranolol

## Abstract

A simple and sensitive method for the determination of propranolol using a modified carbon paste electrode with graphene/Co_3_O_4_ nanocomposite was presented. The electrochemical measurements of propranolol are studied using differential pulse voltammetry, cyclic voltammetry and chronoamperometry. The graphene/Co_3_O_4_ nanocomposite exhibits excellent catalytic activity towards the electrochemical oxidation of propranolol in phosphate buffer solution of pH 7.0. The graphene/Co_3_O_4_ nanocomposite facilitates the determination of propranolol in the concentration range 1.0–300.0 μM and a detection limit and sensitivity of 0.3 μM. and 0.1275 μA/μM were achieved.

## Introduction

Propranolol (I) [1-(isopropylamino)-3-(1-napthyloxy)-2- propanol] is a highly effective and non-selective -adrenergic receptor (-blocker) that is widely used in clinical practices to treat cardiovascular disorders, such as angina pectoris, arrhythmia, myocardial infarction, dysfunctional labor and anxiety. It possesses no autonomous nervous system activity and is completely absorbed from the gastrointestinal tract in healthy volunteers after oral administration. It affects the regulation of blood circulation by reducing the cardiac frequency, myocardial contractility, contraction force, coronary flow and secretion of rennin, resulting in a fall in the level of angiotensin II, which contributes significantly to the hypertensive action of this drug. It also lowers blood pressure by altering the transmission of nerve impulses from the brain to a certain part of the body and causes relaxation (and possibly dilation) of blood vessels, decreasing heart rate and pulse. Propranolol has been listed as a banned substance by World Anti-Doping Agency (WADA) and International Olympic Committee in competitive games. Therefore, monitoring the level of propranolol in biological fluids is important for clinical practices and doping control. To ensure the reliability of results obtained from analytical methods, WADA has established the minimum required performance limit of this drug in urine as 500 g/L [1-5].

There are various methods, such as RP-HPLC-DAD, chromatography, 13C NMR spectroscopic and electrochemical method [5-9]. Among all these quantification techniques, electrochemical techniques offered more sensitivity and reliability in sample consumption and miniaturization of the processes [10-17].

Nanotechnology has enormous potential for providing innovative solutions to a wide range of applications [18-23]. The development of nanoscience and nanotechnology has allowed trials to apply different nanomaterials for the fabrication of chemically modified electrodes [24-36]. In recent years, various nanomaterials have been used singly or in composite form to modify electrodes [37-47]. The chemical modification of inert substrate electrodes offers significant advantages in the design and development of electrochemical sensors [48-52]. In operations, the redox-active sites often shuttle electrons between a solution of the analyte and the substrate electrodes, with a significant reduction of the activation overpotential [53-55]. A further advantage of chemically modified electrodes is that they are less prone to surface fouling and oxide formation compared to inert substrate electrodes [56-60].

The carbon paste electrode (CPE) has attracted great attention in electrochemistry due to its non-toxic nature, eco-friendly, low-cost, easy preparation, broad operational potential window, easy chemical and mechanical modification, and renewable surface. Carbon paste is a widely used electrode due to its electrochemical characteristics, such as very low background current, low ohmic resistance, affordability, easy modification and simple renewal of the electrode surface [61-63].

The objective of the present research is the fabrication of a new sensor by modification of a carbon paste electrode with graphene/Co_3_O_4_ nanocomposite (Gr-Co_3_O_4_ NC/CPE) for determination of the propranolol. Finally, this technique experienced a successful application for detecting propranolol in the real sample with encouraging outputs.

## Experimental

### Equipment and materials

In order to do electrochemical tests at ambient temperature, we utilized the Auto-lab potentiostat/galvanostat (PGSTAT 302N, Eco Chemie, the Netherlands) with GPES (General Purpose Electrochemical System-version 4.9) software to control the system. Electrochemical measurements were performed at room temperature in a conventional electrochemical cell with a Gr-Co_3_O_4_ NC/CPE as the working electrode, 3.0 M Ag/ AgCl/KCl as a reference electrode (Azar Electrode, Urmia, Iran) and platinum wire as a counter electrode (Azar Electrode, Urmia, Iran). Moreover, pH was measured using the Metrohm 713 pH meter with a glass electrode (Switzerland). Propranolol and all other solutions used during the procedure were prepared by reagent-grade chemicals from Merck and Sigma-Aldrich and deionized water was supplied from Millipore, Germany.

### Preparation of graphene/Co_3_O_4_ nanocomposite

**Figure 1.**
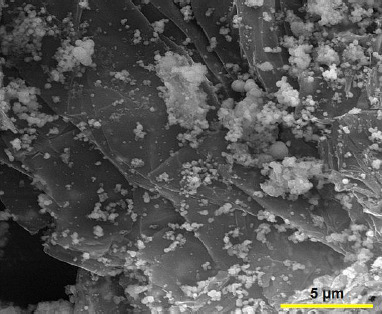
FE-SEM image of graphene/Co_3_O_4_ nanocomposite.

Firstly, graphene oxide (GO) (20 mg) was dispersed in 20 mL ethanol and ultrasonicated for 30 min. Then, Co(NO_3_)_2_.6H_2_O (0.001 mol) was dissolved in 20 mL ethanol solution and stirred for 30 min at ambient temperature. Subsequently, the prepared two solutions were mixed under stirring and 3.6 mL of ammonia solution (NH_3_.H_2_O (wt. 25%)) was dropwise added. The mixture was transferred into a Teflon-lined stainless steel autoclave and maintained at 180 °C for 24 h. After completion of the reaction, the product was collected by centrifugation, and washed with deionized water and ethanol. Finally, the graphene/Co_3_O_4_ nanocomposite was dried at 70 °C overnight in an oven. Figure 1 shows the FE-SEM image *of* graphene/Co_3_O_4_ nanocomposite*.*

### Preparation and surface modification of electrode

**Scheme 1.**
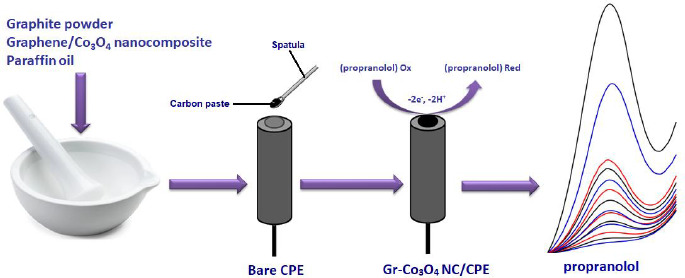
Scheme of Gr-Co_3_O_4_ NC/CPE preparation and voltammetric detection of propranolol.

To prepare Gr-Co_3_O_4_ NC/CPE, 0.95 g graphite powder and 0.05 g graphene/Co_3_O_4_ nanocomposite were mixed. Next, a suitable amount of paraffin oil was poured into the resulting mixture, followed by mixing well for 30 min to obtain a uniformly wetted paste. An appropriate amount of the paste was tightly packed into a glass tube and a copper wire was positioned over the carbon paste to make electrical contact (Scheme 1).

The surface areas of the Gr-Co_3_O_4_ NC/CPE and the un-modified CPE were obtained by CV using 1 mM K_3_Fe(CN)_6_ at various scan rates. Using the Randles–Ševčik equation for Gr-Co_3_O_4_ NC/CPE, the electrode surface was 0.342 cm2, about 3.8 times greater than un-modified CPE.

## Results and discussion

### Electrochemical behavior of propranolol on graphene/Co_3_O_4_ nanocomposite

**Figure 2.**
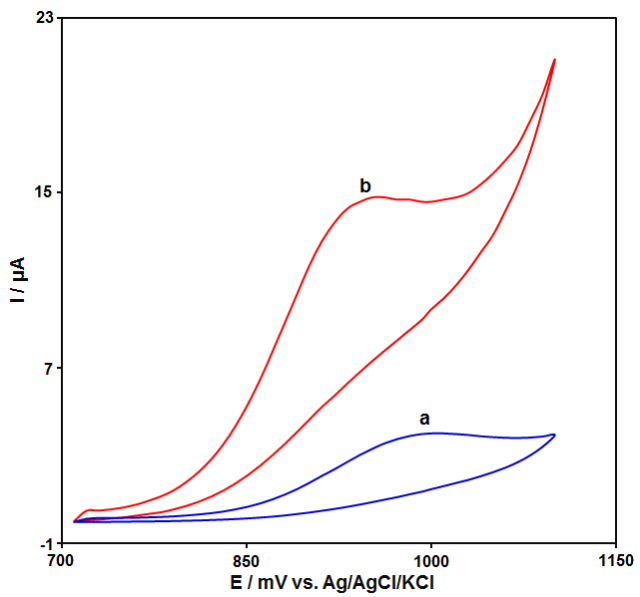
Cyclic voltammograms of a) CPE and b) Gr-Co_3_O_4_ NC/CPE in the presence of 100.0 μM propranolol at a pH 7.0 of 0.1 M PBS, respectively.

The electrochemical behavior of the CPE, Gr-Co_3_O_4_ NC/CPE was studied by the cyclic voltammetry (CV) technique in the 0.1 M phosphate buffer (pH 7.0) as the supporting electrolyte at a scan rate of 50 mV s^−1^ (Figure 2). As shown in Figure 2, in comparison to the bare CPE (a), Gr-Co_3_O_4_ NC/CPE (b) presents a well-defined Irreversible oxide peak with a higher current signal (propranolol concentration equal to 100.0 μM).

### Role of variable scan rates

**Figure 3.**
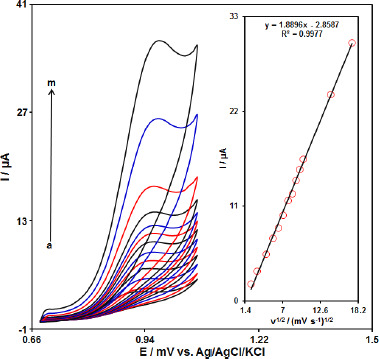
Cyclic voltammograms of propranolol (70.0 μM) at Gr-Co_3_O_4_ NC/CPE at different scan rates of a) 5, b) 10, c) 20, d)30, e) 40, f) 50, g) 60, h) 70, i) 80, j) 90 k) 100 l) 200 and m) 300 mV/s in 0.1 M PBS (pH 7.0). Insert: Plot of *I*p versus *v* ^1/2^ for the oxidation of propranolol at Gr-Co_3_O_4_ NC/CPE.

The effect of the potential scan rates (5-300 mV s^-1^) on the electrochemical oxidation of propranolol was studied by cyclic voltammetry (CV). Figure 3 shows the CV of 70.0 μM of propranolol in the 0.1 M phosphate buffer solution at the Gr-Co_3_O_4_ NC/CPE. These results show that the anodic current increases with an increasing scan rate. The oxidation current of propranolol increased linearly with the square root of the scan rate (Figure 3, Inset), which demonstrate a diffusion controlled electrochemical process.

### Chronoamperometric analysis

**Figure 4.**
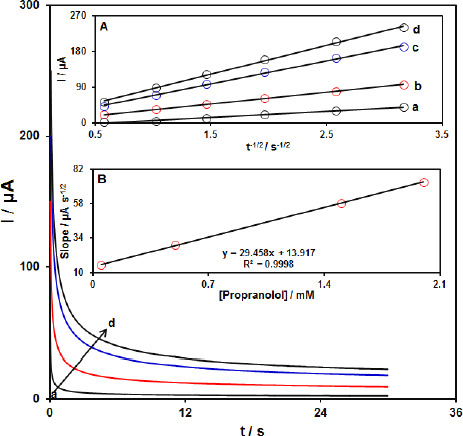
Chronoamperograms obtained at the Gr-Co_3_O_4_ NC/CPE in the presence of a) 0.05, b) 0.5, c) 1.5 and d) 2.0 mM propranolol in the 0.1 M buffer solution (pH 7.0). A) Plot of *I* versus *t*^-1/2^ for electro-oxidation of hydrochlorothiazide obtained from chronoamperograms a–d. B**)** Plot of slope from straight lines versus propranolol level.

The chronoamperometric measurements of propranolol at the Gr-Co_3_O_4_ NC/CPE surface were done to estimate the apparent diffusion coefficient of propranolol under used experimental conditions. Figure 4 shows the current-time profiles obtained by setting the working electrode potential at 1000 mV for different concentrations of propranolol. At long enough experimental times (*t*=0.3-3s), where the electron transfer reaction rate of propranolol is more than its diffusion rate toward the working electrode surface, the current is diffusion controlled. Figure 4, inset A, shows the experimental plots of *I* versus *t*^-1/2^ with the best fit for different concentrations of propranolol employed. The slopes of the resulting straight lines were then plotted versus the propranolol concentration (Figure 4, inset B). Based on the Cottrell equation [64], the slope of this plot (Figure 4 inset B) can be used to estimate the apparent diffusion coefficient of propranolol. From the slope of this plot (29.458 A s^1/2^ mM^-1^), the value of propranolol was found to be 7.4x10^-5^ cm s^1^ for propranolol.

### Differential pulse voltammetry analysis of propranolol

**Figure 5.**
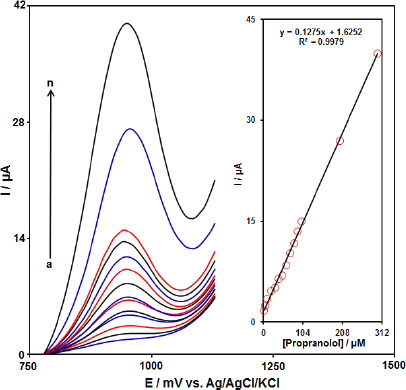
DPV curves of Gr-Co_3_O_4_ NC/CPE in the0.1 M buffer solution (pH 7.0) containing different concentrations of propranolol. a-n corresponds to 1.0, 5.0, 10.0, 20.0, 30.0, 40.0, 50.0, 60.0, 70.0, 80.0, 90.0, 100.0, 200.0 and 300.0 μM propranolol. Inset: Plots of electrocatalytic peak current as a function of propranolol concentration.

Differential pulse voltammetry (DPV) was used for the determination of propranolol at Gr-Co_3_O_4_ NC/CPE due to its high sensitivity. The DPV responses for different concentrations of propranolol were illustrated in Figure 5 (step potential=0.01 V and pulse amplitude=0.025 V). The linear range was found to be from 1.0 μM to 300.0 μM. The linear equation was *I*_p_ (μA)=1.6252+0.1275 *C*_propranolol_ (μM) with a correlation coefficient of 0.9979. Also, the limit of detection, *C*_m_, of propranolol was calculated using the following equation:



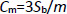



where, *m* is the slope of the calibration plot (0.1275 μA/ μM) and *S*_b_ is the standard deviation of the blank response obtained from 10 replicate measurements of the blank solution. The detection limit for the determination of propranolol using this method is 0.3 μM.

## Conclusion

We arranged a sensor using Gr-Co_3_O_4_ nanocomposite produced for the detection of propranolol and showed that the results showed the improved behavior of the propranolol sensor with respect to the nanocomposite by s-created. Extensive direct access, limited detection limit (0.3 μM), selectivity and incredible robustness were found for the proposed detection framework as a result of improved properties such as large surface area, fast mass vehicle, remarkable biocompatibility and reactive synergism of Gr-Co_3_O_4_ nanoparticles. Likewise, the findings recommended Gr-Co_3_O_4_ NC/CPE nanoparticles as a magnificent sensor for the determination of propranolol.
